# WNT Inhibitory Activity of *Malus Pumila* *miller* cv Annurca and *Malus domestica* cv Limoncella Apple Extracts on Human Colon-Rectal Cells Carrying Familial Adenomatous Polyposis Mutations

**DOI:** 10.3390/nu9111262

**Published:** 2017-11-18

**Authors:** Gennaro Riccio, Maria Maisto, Sara Bottone, Nadia Badolati, Giovanni Battista Rossi, Gian Carlo Tenore, Mariano Stornaiuolo, Ettore Novellino

**Affiliations:** 1 Department of Pharmacy, University of Naples Federico II, 80131 Naples, Italy; genriccio@gmail.com (G.R.); maria.maisto@unina.it (M.M.); sara.bottone@unina.it (S.B.); badolatin@gmail.com (N.B.); giancarlo.tenore@unina.it (G.C.T.); 2 Gastroenterology and Gastrointestinal Endoscopy Unit, Istituto Nazionale Tumori-IRCCS-Fondazione G. Pascale, 80131 Naples, Italy; g.rossi@istitutotumori.na.it

**Keywords:** nutraceuticals, apple polyphenols, WNT pathway inhibitors, familial adenomatous polyposis, colon cancer

## Abstract

Inhibitors of the Wingless-related Integration site (WNT)/β-catenin pathway have recently been under consideration as potential chemopreventive agents against Familial Adenomatous Polyposis (FAP). This autosomal-dominant syndrome is caused by germline mutations in the gene coding for the protein APC and leads to hyperactivation of the WNT/β-catenin signaling pathway, uncontrolled intestinal cell proliferation and formation of adenocarcinomas. The aim of the present work was to: (i) test, on in vitro cultures of cells carrying FAP mutations and on ex vivo biopsies of FAP patients, the WNT inhibitory activity of extracts from two common southern Italian apples, *Malus pumila Miller* cv. ‘Annurca’ and *Malus domestica* cv ‘Limoncella’; (ii) identify the mechanisms underpinning their activities and; (iii) evaluate their potency upon gastrointestinal digestion. We here show that both Annurca and Limoncella apple extracts act as WNT inhibitors, mostly thanks to their polyphenolic contents. They inhibit the pathway in colon cells carrying FAP mutations with active dilutions falling in ranges close to consumer-relevant concentrations. Food-grade manufacturing of apple extracts increases their WNT inhibitory activity as result of the conversion of quercetin glycosides into the aglycone quercetin, a potent WNT inhibitor absent in the fresh fruit extract. However, in vitro simulated gastrointestinal digestion severely affected WNT inhibitory activity of apple extracts, as result of a loss of polyphenols. In conclusion, our results show that apple extracts inhibit the WNT pathway in colon cells carrying FAP mutations and represent a potential nutraceutical alternative for the treatment of this pathology. Enteric coating is advisable to preserve the activity of the extracts in the colon-rectal section of the digestive tract.

## 1. Introduction

Familial adenomatous polyposis (FAP) is an inherited gastrointestinal syndrome, characterized by the formation of a large number of adenomas throughout the small intestine and the large bowel. FAP has a birth incidence of about 1/8300, manifests equally in both sexes, and accounts for less than 1% of colorectal cancer (CRC) cases [1]. The birth frequency of FAP patients in European populations is estimated at roughly 1 in 11,300–37,600 live births and its progression to colorectal cancer is close to 100% by the age of 35–40 years [2]. While colectomy remains the optimal prophylactic treatment, the identification of chemopreventive agents represents one of the major challenges for the future [2].

FAP is caused by a germline mutation in the *Adenomatous Polyposis Coli (APC)* gene on chromosome 5q21–q22. This locus contains a tumor suppressor gene encoding for the protein APC, that functions intracellularly as a scaffold in a large protein complex, known as “β-catenin destruction complex” [3]. This includes the serine/threonine kinase, glycogen synthase kinase-3 β (GSK-3β), Axin, and casein kinase I [4]. The complex represents an important intracellular checkpoint. In virtue of its ability to target β-catenin for proteasomal degradation, it reduces its intracellular levels. This activity avoids β-catenin translocation into the nucleus, binding to the transcription factors, TCF and LEF, and induction of oncogenes, like *c-myc* and *cyclin D1* [5,6].

In wild type cells, APC counterbalances the activity of the Wingless-related Integration site (WNT) pathway, a signaling cascade regulating development in embryos and tissue homeostasis in adult organs. In the gastrointestinal (GI) tract, WNT supports the self-renewal capacity of epithelial stem cells and allows GI organs to be the most intensively self-replenishing in mammals [7]. The class F G-Protein-Coupled Receptor (GPCR) family members, Frizzleds (FZDs), act as a WNT receptors. Upon activation, these recruit and disassemble the β-catenin destruction complex, inhibiting its function and causing β-catenin intracellular accumulation and nuclear translocation [5]. This is the reason for why FAP mutations, by abolishing APC function, lead to constitutively active WNT signaling and, in turn, to uncontrolled proliferation of colon cells, formation of polyps and adenocarcinomas [8].

Apple extracts have been shown to mediate several biological cellular effects that might be of interest with respect to chemoprevention of colorectal diseases [9,10,11,12]. Such activity mostly relies on the high number of polyphenols they contain [13,14,15]. Polyphenol-rich apple extracts have been shown to suppress human colon cancer cell growth in several in vitro culture models [16,17,18]. Moreover, in *APC^Min/+^* mice (a murine model of FAP), the consumption of beverages containing apple polyphenol extracts has been shown to affect the number and growth of colon polyps and reduce colorectal bleeding and high-grade dysplasia [19]. So far, the biological activity of apple polyphenols has been mainly ascribed to their antioxidant potential [20]. However, the exact mechanisms underpinning WNT’s inhibitory activity of apple extract is not yet clear. Recently, several polyphenols have been proposed of being endowed with modulatory activities toward specific protein targets; this includes, among others, many of the components WNT signaling pathway [11,14,21].

The aim of the present work was to test the WNT inhibitory activity of two apple cultivars, native to Southern Italy, namely “Annurca” and “Limoncella”, identify the polyphenols mainly responsible for their inhibitory activity and determine their mechanism of action.

*Malus pumila Miller* cv. Annurca is a widespread apple and accounts for 5% of Italian apple production. It is listed as a Protected Geographical Indication (PGI) product from the European Council (Commission Regulation (EC) No. 417/2006)). This apple has been already shown to possess nutraceutical potential in virtue of its ability to reduce cellular glucose levels and lipid uptake [22,23,24,25]. *Malus domestica* cv ‘Limoncella’ is a juicy and aromatic variety of apple, known since ancient Roman times [26]. It is resistant to long time storage and can survive cold winters. In contrast to Annurca, Limoncella’s nutraceutical potential has not yet been documented [27].

In the current study, we tested, on in vitro cultures of cells carrying FAP mutations and on ex vivo biopsies of FAP patients, the WNT inhibitory activity of Annurca and Limoncella apple extracts, aimed to identify the mechanism underpinning their activity and evaluate their potency upon in vitro simulated GI digestion.

## 2. Materials and Methods

### 2.1. Reagents

Chemicals and reagents used were either analytical-reagent or HPLC grade. The water was treated in a Milli-Q water purification system (Millipore, Bedford, MA, USA) before use. The standards used for the identification and quantification of phenolic acids and flavonoids were chlorogenic acid, (+)-catechin, (−)-epicatechin, phloretin, phloridzin, procyanidin B2, quercetin, rutin (quercetin-3-*O*-rutinoside) and isoquercetin (quercetin-3-*O*-glucoside), (Sigma Chemical Co., St. Louis, MO, USA). Chemicals and reagents used to simulate the GI digestion were potassium chloride (KCl), potassium thiocyanate (KSCN), monosodium phosphate (NaH_2_PO_4_), sodium sulphate (Na_2_SO_4_), sodium chloride (NaCl), sodium bicarbonate (NaHCO_3_), urea, α-amylase, hydrochloric acid (HCl), pepsin, pancreatin and bile salts (Sigma Chemical Co., St. Louis, MO, USA). Acetonitrile and methyl alcohol were of HPLC grade (Carlo Erba, Milano, Italy). The Nuclear Factor of Activated T-cells (NFAT) inhibitor, cell permeable (sequence: RRRRRRRRRRRGG GMAGPHPVIVITGPHEE; #592517-80-1, Tocris, Bristol, UK) was dissolved in water; Bisindolymaleimide II (3-(1H-Indol-3-yl)-4-[1-[2-(1-methyl-2-pyrrolidinyl)ethyl]-1H-indol-3-yl]-1H-pyrrole-2,5-dione, #137592-45-1, Tocris, Bristol, UK) was dissolved in DMSO.

### 2.2. Fruit Collection and Sample Preparation

Annurca (*Malus Pumila Miller* cv Annurca) apple fruits and Limoncella (*Malus Domestica* cv Limoncella) were collected from Valle di Maddaloni (Caserta, Italy), in October 2016, when fruits had just been harvested. Annurca fruits were reddened for about 30 days [23], and then analyzed. Lyophilised peels and flesh (10 g) of Limoncella and Annurca apple samples were treated with 60 mL of 80% methanol (0.5% formic acid), homogenized for 5 min by ultra-turrax (T25-digital, IKA, Staufen im Breisgau, Berlin, Germany) and shaken on an orbital shaker (Sko-DXL, Argolab, Carpy, Italy) at 300 rpm for 15 min. Then, the samples were placed in an ultrasonic bath for another 10 min, before being centrifuged at 6000 rpm for 10 min. The supernatants were collected and stored in darkness, at 4 °C. The pellets obtained were re-extracted, as described above and with another 40 mL of the same mixture of solvents. Finally, the extracts obtained were filtered under vacuum, the methanol fraction was eliminated by evaporation, and the water fraction was lyophilized. To obtain polyphenol-enriched fractions of Annurca apple extract (AAE) and Limoncella apple extract (LAE) (in the text referred to as PEF(AAE) and PEF(LAE), respectively) the dry extracts were dissolved in distilled water and slowly filtered through an Amberlite XAD-2 column, packed as follows: Resin (10 g; pore size 9 nm with particle sizes of 0.3–1.2 mm; Supelco, Bellefonte, PA, USA) was soaked in methanol, stirred for 10 min and then packed into a glass column (10 × 2 cm). The column was washed with 100 mL of acidified water (pH 2) and 50 mL of deionized water for removal of sugars and other polar compounds. The adsorbed phenolic compounds were extracted from the resin by elution with 100 mL of methanol, which was evaporated by flushing with nitrogen. The extracts were stored at −80 °C until HPLC analysis. Before performing the biological tests, each extract was dissolved in DMSO at a final concentration of 300 mg/mL. Food grade Limoncella apple extracts (IndLAE) were produced at MB-Med (Turin, Italy) starting from fresh Limoncella Apples. Upon lyophilization of peels and flesh of Limoncella apples, samples were treated with ethanol/water (70:30 *v*/*v*) for 24 h at 40 °C to extract phenolic compounds and generate food grade Limoncella Apple Extracts (in the text referred to as IndLAE).

### 2.3. In Vitro Simulated GI Digestion of Apple Extracts

The assay was performed according to the procedure described by Raiola et al. [28] and by Tenore et al. [23], with few modifications. For GI digestion, the apple samples (20 g) were mixed with 6 mL of artificial saliva composed of KCl (89.6 g/L), KSCN (20 g/L), NaH_2_PO_4_ (88.8 g/L), Na_2_SO_4_ (57.0 g/L), NaCl (175.3 g/L), NaHCO_3_ (84.7 g/L), urea (25.0 g/L) and 290 mg of α-amylase. The pH of the solution was adjusted to 6.8 with HCl 0.1 N. The mixture was introduced in a plastic bag containing 40 mL of water and homogenized in a Stomacher 80 Microbiomaster (Seward, Worthing, UK) for 3 min. Immediately, 0.5 g of pepsin (14,800 U) dissolved in HCl 0.1 N was added, the pH was adjusted to 2.0 with HCl 6 N, and the solution was incubated at 37 °C in a Polymax 1040 orbital shaker (250 rpm) (Heidolph, Schwabach, Germany) for 2 h. Then the pH was increased to 6.5 with NaHCO3 0.5 N and 5 mL of a mixture of pancreatin (8.0 mg/mL) and bile salts (50.0 mg/ mL) (1:1; *v*/*v*), dissolved in 20 mL of water, was added and incubated at 37 °C in an orbital shaker (250 rpm) for 2 h. Supernatants were extracted with an acetonitrile/water (84:16; *v*/*v*) mixture, and then either analyzed by HPLC or dried and dissolved in DMSO for biological tests.

### 2.4. HPLC-DAD Analysis

HPLC separation and quantification of phenolic compounds in apple extracts and samples obtained from in vitro digestion, were performed according to earlier studies [23] with some modifications. Identification was possible by comparing spectra and retention times with those of commercial standards and with those reported in previous works [29]. The column selected was a Hypersil BDS C18 column (250 mm, 4.6 mm, 5 μm) (Thermo, Bellefonte, PA, USA). Analyses were run on a Finnigan HPLC system (Thermo Electron Corporation, San Jose, CA, USA) provided with a photodiode array detector (DAD). The identity of phenolic acids and flavonoids was confirmed with LC-ESI/MS/MS experiments, as already reported [24].

### 2.5. Cell Cultures

HEK293, CaCo-2, and U87MG cells were grown in DMEM (#41965-039, GIBCO, Thermo Fisher Scientific, Waltham, MA, USA) supplemented with 10% FBS (#10270, GIBCO), Glutamax (#35050-061, GIBCO) and Pen/Strep (#15070-063, GIBCO). HEK293 transfection was performed using Polyethylenimine ( Sigma Chemical Co., St. Louis, MO, USA). CaCo-2 transfection was performed using Lipofectamine (Thermo Fisher Scientific, Waltham, MA, USA) following the manufacturer’s instructions. Both cell cultures and human biopsies were analyzed for viability using Trypan blue, Propidium Iodide (PI) and Acridine-Orange (AO) staining. A pre-mixed AO/PI solution was directly added to cell samples for viability analysis, using a fluorescent cell counter.

### 2.6. Excision and Culturing of Human Biopsies from FAP Patients

Biopsies from Familial Adenomatosis Poliposis patients were kindly provided by Prof. G.B. Rossi. The study was approved by the Ethics Committee of the University Federico II of Naples. For all the patients enrolled in this study, polypectomy was part of their clinical treatment plan and it was scheduled and performed independently from this research. Immediately after excision, biopsies were rinsed in physiological saline. Samples were then digested with Trypsin for 10 min at room temperature (RT) and centrifuged at 400 rpm for 10 min at RT. Isolated cells were then counted and cultured at the confluency of 100,000 cell per mL in DMEM, supplemented with 10% FBS, Glutamax and Pen/Strep. When indicated, apple extracts were added at a concentration of 400 mg/L. After 24 and 48 h of incubation, cell viability was measured with using Trypan blue, Propidium Iodide (PI) and Acridine-Orange (AO) staining.

### 2.7. DNA

All DNA constructs were synthesized at GeneScript (Piscataway, NJ, USA). The cDNA coding for N terminally HA tagged FZD4wt (HA-FZD4-wt), was cloned in the expression vector, pCDNA3.1 (Invitrogen). For the reporter construct, WNT reporter Green Fluorescent Protein GFP construct (TCF-wt GFP), 8 repeats of the optimized TCF/LEF binding sequence [5′-AGATCAAAGGGG-3′] (interspaced by the triplet 5′-GTA-3′) were positioned upstream to a minimal TATA box promoter [5′-tagagggtatataatggaagctcgaattccag-3′] and a KOZAC region [5′-cttggcattccggtactgttggtaaaaagcttggcattccggtactgttggtaaagccacc-3′]. The sequence was cloned in the vector pCDNA 3.1 (+) GFP between the restriction sites for NruI and HindIII. This replacement substitutes the cytomegalovirus (cmv) promoter of the original vector with the TCF/LEF responsive sequences. The correctness of the sequences were verified by DNA sequencing. The reporter construct (TCF-mut GFP) was used as a negative control. This was obtained by the mutagenesis of TCF-wt GFP and presented the 8 repeats of the TCF/LEF binding sequence mutated to [5′-AGGCCAAAGGGG-3′]. The reporter construct, cmv GFP, used in this manuscript as the transfection control, was obtained by replacing the TCF/LEF consensus region in TCF-wt GFP with a cmv promoter.

### 2.8. WNT Pathway Activity Measurement in HEK293 Cells Using the TCF-GFP Constructs

HEK293 cells were seeded (5 × 10^3^ per well) in 96-well black Optyplates (Perkin Elmer, Waltham, MA, USA). After 24 h, cells were co-transfected with both WNT reporter GFP construct and HA-FZD4-wt. Transfection mixtures were prepared as follows: for each well, 0.25 µg of PEI (pH 7.0) was supplemented with 0.08 µg of DNA (both diluted in 4 μL of DMEM). The mixture was incubated at room temperature (RT) for 30 min, to be then diluted in culture medium and added to the cells. Twenty-four hours after transfection, the medium was replaced and cells were incubated with WNT5A conditioned medium. Human glyoblastoma U87MG cells were used as a source of WNT5A conditioned medium [30]. U87MG cells endogenously express FZD4, which has been shown to be necessary for the activity of WNT/β-catenin pathway in these cells [31]. Confluent 6 cm plates of U87MG cells were incubated for 3 days in DMEM, 10% FBS and Glutamax, in the absence of antibiotics. Conditioned medium was subsequently collected and stored at −20 °C. Cells were then rinsed with fresh medium and cultivated for a further period of 3 days. The conditioned medium obtained after the second incubation was pooled with the first one. The pooled conditioned medium was used to stimulate TCF/LEF activity in HEK293 cells. It was used undiluted. When required, the conditioned medium was supplemented with apple extracts at the indicated concentrations. When reported, cells were supplemented with LiCl, Epidermal Growth Factor (EGF), NFAT inhibitor (10 µM) or Bisindolylmaleimide II (7.5 µM). After 48 h, cells were fixed in 3.7% formaldehyde, in PBS (pH 7.4), for 30 min. Formaldehyde was quenched by incubating the cells for 30 min in 0.1 M glycine in PBS 1x. The activity of the apple extract was evaluated by measuring GFP expression.

### 2.9. WNT Pathway Activity Measurement in CaCo-2 Cells Using the TCF-GFP Constructs

CaCo-2 cells, growing on glass coverslips, were transfected by Lipofectamine , according to the to the manufacturer’s procedures. Twenty-four hours after transfection, the medium was replaced and cells were incubated with the indicated extracts for 24–48 h. The cells were then fixed in a solution of 3.7% formaldehyde, in PBS with a pH 7.4, for 30 min. Formaldehyde was quenched by incubating the cells for 30 min in 0.1 M glycine in PBS.

### 2.10. Immunofluorescence

CaCo-2 cells were grown on glass coverslips. Cells were fixed in 3.7% formaldehyde/PBS (pH 7.4) for 30 min. Formaldehyde was quenched by incubating the cells for 30 min in 0.1 M glycine in PBS 1x. Then, cells were permeabilized in 0.1% Triton/PBS, pH 7.4, for 10 min at 25 °C and then incubated with a rabbit polyclonal anti-β-catenin antibody (H-102, sc-7199, Santa Cruz, Dallas, TX, USA) diluted 1:200 in PBS and a goat anti-rabbit IgG (H&L), DyLight 594 conjugate, (ImmunoReagents, Raleigh, NC, USA) (diluted 1:500 in PBS), for 1 h and 45 min, respectively.

### 2.11. Statistical Analysis

Unless otherwise stated, all of the experimental data are shown as mean ± standard deviation (SD) of at least three replications [32]. Statistical analyses of data were performed using the Student’s *t*-test or two-way ANOVA, followed by the Tukey–Kramer multiple comparison test, to evaluate significant differences between a pair of means. For dose-response data, half maximal effective concentrations (EC_50_) were calculated using nonlinear regression analysis with Prism software 6.0 (GraphPad, GraphPad Software, San Diego, CA, USA, www.graphpad.com). EC_50_ are indicated as mean ± standard error mean (SEM). All data were analyzed using the two-tailed Student’s *t*-test and *p* < 0.05 indicated a statistically significant result.

## 3. Results

### 3.1. WNT Inhibitory Activity of AAE and LAE

The total (flesh and peel) polyphenolic content in AAE and LAE prepared for this study was determined by HPLC-DAD analysis and is reported in Table 1. AAE and LAE presented a qualitatively similar polyphenolic repertoire.

They contained a similar amount of chlorogenic acid, the most abundant hydroxycinnamic acid in apples, as well as of the flavan-3-ols, (+) catechin and (–) epicatechin. The quercetin-glycoside, rutin and the phloretin–glycoside, phloridzin, were more abundant in AAE than in LAE. The latter, on the contrary, contained higher amounts of procyanidin B2 and isoquercetin [24,26]. The aglycones, quercetin and phloretin were absent in both the extracts.

The activity of the apple extracts on WNT/β-catenin signaling was assayed in a reconstituted recombinant system. We used, as a biological platform, human embryonic HEK293 cells, transiently expressing both the WNT receptor Frizzled 4 (FZD4) [33] and a WNT pathway reporter DNA construct. Three different WNT reporter constructs were used. The first, hereinafter referred to as TCF-wt GFP, presents the coding sequence of GFP under the control of an optimized WNT pathway responsive promoter. In the second, hereinafter referred to as TCF-mut GFP, the WNT responsive promoter was mutagenized to become unresponsive to WNT (see methods for details). Finally, a third reporter construct (cmv GFP) presents the coding sequence of GFP under the control of a constitutive cmv promoter. The TCF-mut GFP and cmv GFP constructs were here considered as negative controls, and used to monitor WNT unrelated change in GFP expression.

The FZD agonist, WNT5A, induced GFP expression in HEK293 cells, transiently transfected with TCF-wt GFP (Figure S1). On the contrary, WNT5A did not affect GFP expression in cells transfected either with TCF-mut GFP or with cmv GFP (Figure S1), confirming the specificity of the platform. At the endogenous level, HEK293 cells express several other FZD receptors. However, differently from FZD4, these did not respond to WNT5A stimulation. As shown in Figure S1, in the absence of FZD4, WNT5A stimulation does not influence GFP expression in HEK293.

WNT5A has been shown to activate both the WNT/β-catenin pathway as well as one of the “non-canonical” branches of WNT signaling, the WNT/Ca^2+^ pathway [5]. In our biological system, WNT5A increased GFP expression, mainly as a consequence of WNT/β-catenin pathway activation.

Inhibitors of Nuclear Factor of Activated T-cells (NFAT) and Protein Kinase C (PKC) (both key elements of the WNT/Ca^2+^ pathway) did not affect GFP expression induced by WNT5A (Figure S1). The combination of WNT5A and a recombinantly expressed FZD4 allowed us a clear interpretation of the effect of apple extracts on the WNT/β-catenin signaling pathway.

**Figure 1:**
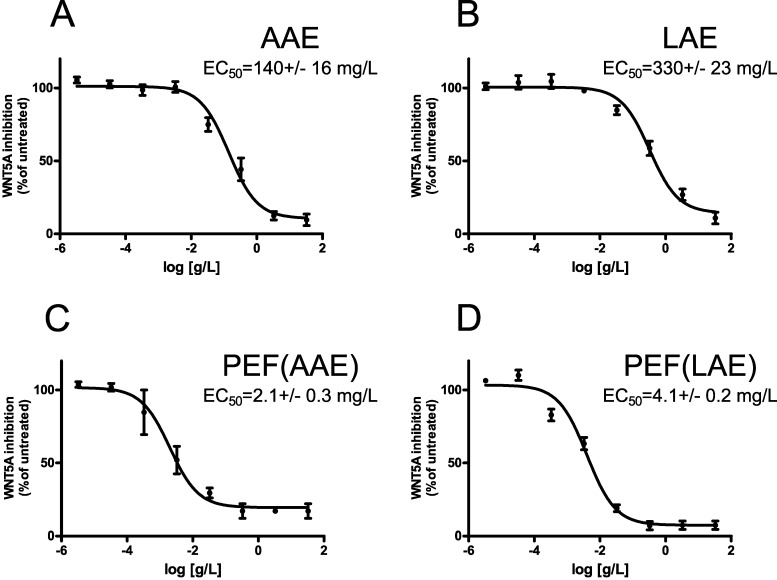
AAE and LAE act as WNT pathway inhibitors. (**A**,**B**) Dose-response curves represent AAE (**A**) and LAE (**B**) modulation of WNT/β-catenin pathway in HEK293 cells co-expressing FZD4 and the WNT reporter construct (TCF-wt GFP). Values indicate changes in GFP expression (expressed as mean percentage change over untreated samples). (**C**,**D**) Dose-response curves represent PEF(AAE) (**C**) and PEF(LAE) (**D**) modulation of WNT/β-catenin pathway. Values are reported as mean ± SD (*n* = 3 replicates). EC_50_ values for each sample are shown on the corresponding graph and are reported as mean ± SEM (*n* = 5 independent experiments).

AAE and LAE both worked efficiently as WNT inhibitors and reduced WNT activity elicited by WNT5A. The EC_50_ of WNT pathway inhibition were 140 ± 16 mg/L and 330 ± 23 mg/L for AAE and LAE, respectively (Figure 1A,B). AAE and LAE did not affect GFP expression in cells transfected either with TCF-mut GFP or with cmv GFP (Figure S2), confirming that the two extracts affect the WNT/β-catenin pathway.

The WNT inhibitory activity of both the extracts mainly depends on their polyphenolic content. Compared to total fresh fruit extracts, polyphenolic enriched fractions of AAE and LAE, (hereinafter indicated as PEF(AAE) and PEF(LAE)) (Table 1) were associated with increased WNT inhibitory activity (2.1 ± 0.3 mg/L and 4.1 ± 0.2 mg/L for PEF(AAE) and PEF(LAE), respectively) (Figure 1C,D). The WNT inhibitory activity of both full extracts and polyphenolic fractions cannot be ascribed to their toxicity, which occurs at dilutions higher than 5.0 g/L (Figure S2).

### 3.2. WNT Inhibitory Activity of Pure Polyphenols

**Figure 2:**
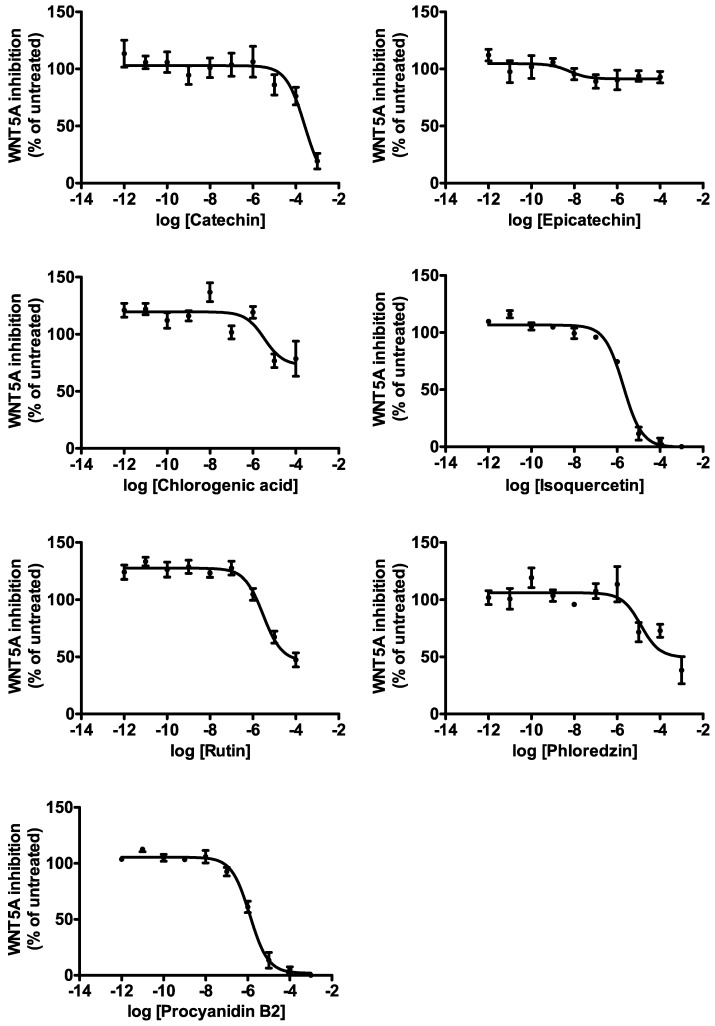
WNT inhibitory activity of apple polyphenols. Dose-response curves represent the indicated compounds modulation of WNT pathway in HEK293 cells. Values are reported as mean ± SD (*n* = 3). EC_50_ values for each compound are reported in Table 2.

In an attempt to identify which of the polyphenols contained in AAE and LAE were mostly contributing to the WNT inhibitory activity of the apple extracts, we tested them singularly, as pure molecules (Figure 2 and Table 2). HPLC grade, pure epicatechin and catechin both showed very weak WNT5A inhibitory activities. Chlorogenic acid had a WNT inhibitory activity of 3.4 ± 0.2 μM. Rutin and isoquercetin inhibited WNT5A with EC_50_ of 1.8 ± 0.1 μM and 3.1 ± 0.4 μM, respectively. Phloridzin and procyanidin B2 showed EC_50_ of 12.9 ± 0.5 μM and 1.4 ± 0.3 μM, respectively. Considering their low abundance in both the apple extracts, none of the abovementioned molecules would reach effective concentrations in AAE and LAE solutions diluted at their EC_50_ for WNT inhibition (Table 2). Thus, while polyphenols surely contribute to the WNT inhibitory activityof the apple extracts, none of the polyphenols by themselves can account for the full activity of AAE and LAE, which seem to require, on the contrary, the presence of the whole polyphenolic fraction.

### 3.3. Mechanism Underpinning AAE and LAE WNT Inhibitory Activity

We thus moved to identifying the WNT pathway branches inhibited by AAE and LAE. This is very important, especially when searching for therapeutic agents to use for FAP patients. Since APC is a midstream component of the WNT pathway, its mutations make the manipulation of most of the upstream signaling components therapeutically ineffective. To be active in FAP patients, WNT inhibitors should act either downstream to APC or on “non-canonical” WNT pathway branches. One of the most active non-canonical branches positively contributing to WNT signaling is the one involving the EGF Receptor (EGFR) [34,35]. Once activated, the EGFR pathway bypasses APC and leads, via AKT, to β-catenin activation.

**Figure 3:**
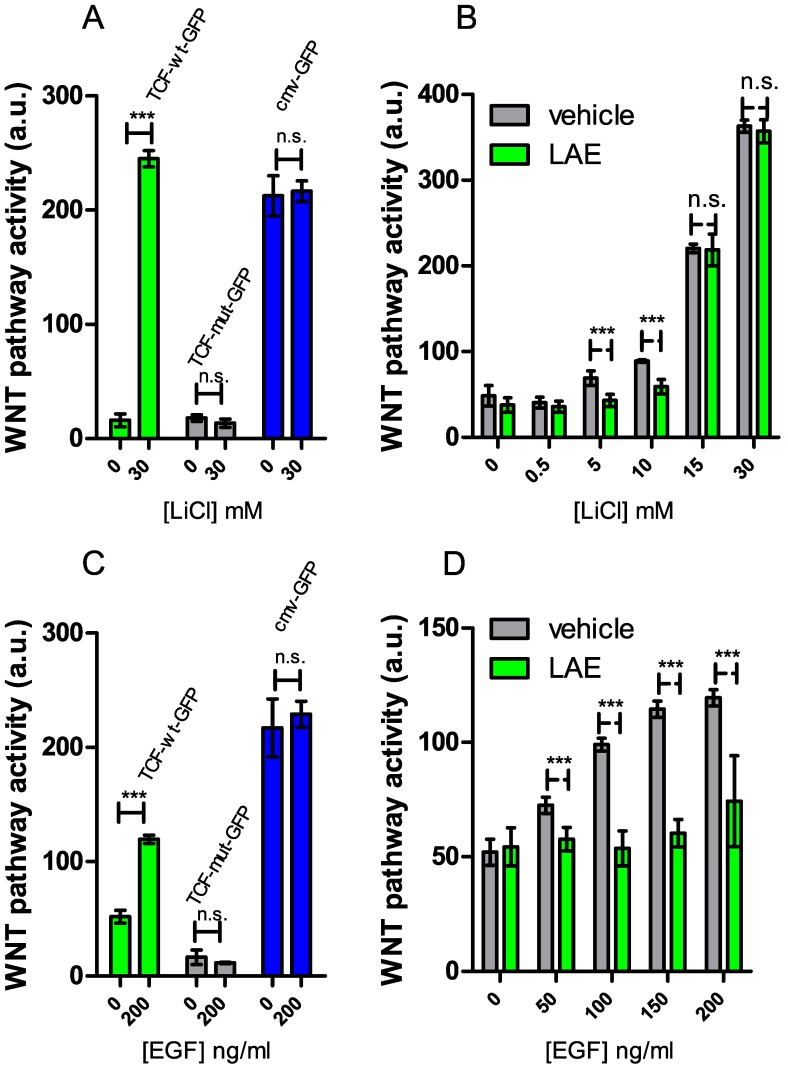
LAE inhibits the WNT pathway, acting downstream to APC. (**A**) The histogram shows the WNT pathway activity induced by LiCl (30 mM) in HEK293 cells transfected with TCF-wt GFP (green bars), TCF-mut GFP (grey bars) and cmv GFP (blue bars); (**B**) WNT pathway activity of cells treated with the indicated concentration of LiCl in the presence (green bars) or in the absence (grey bars) of LAE (400 mg/L); (**C**) WNT pathway activity induced by EGF (200 ng/mL) in HEK293 cells transfected with TCF-wt GFP (green bars), TCF-mut GFP (grey bars) and cmv GFP (blue bars); (**D**) WNT pathway activity of cells treated with the indicated concentration of EGF in the presence (green bars) or in the absence (grey bars) of LAE (400 mg/L). Values are reported as mean ± SEM (*n* = 5). *** *p* < 0.05, n.s. indicates non-statistical.

We thus challenged AAE and LAE to compete with LiCl and EGF, two inducers of the WNT pathway, both acting downstream to APC. LiCl binds directly to GSK-3β and inhibits the β-catenin destruction complex. In contrast, EGF activates the EGFR pathway, that, via AKT, promotes β-catenin detachment from the Plasma Membrane and its nuclear translocation. In our biological system, both LiCl and EGF induced, in a dose-response manner, GFP expression in HEK293 transfected with TCF-wt GFP (Figure 3A–C). On the contrary, they both did not have an effect on cells transfected either with TCF-mut GFP or with cmv GFP (Figure 3A–C).

AAE and LAE failed to inhibit activation of the WNT pathway induced by 15 and 30 mM LiCl. However, they both reduced WNT pathway activity induced by 5 and 10 mM LiCl (the results for LAE are depicted in Figure 3B). Moreover, at all the tested concentrations, the extract abolished the WNT stimulatory activity of EGF [34] (Figure 3D). These results prove that the apple extracts inhibit WNT pathway activation induced by LiCl and EGF and are thus suitable WNT inhibitors for FAP cells carrying APC mutations.

### 3.4. WNT Inhibitory Activity of LAE and AAE on CaCo-2 Cells

**Figure 4:**
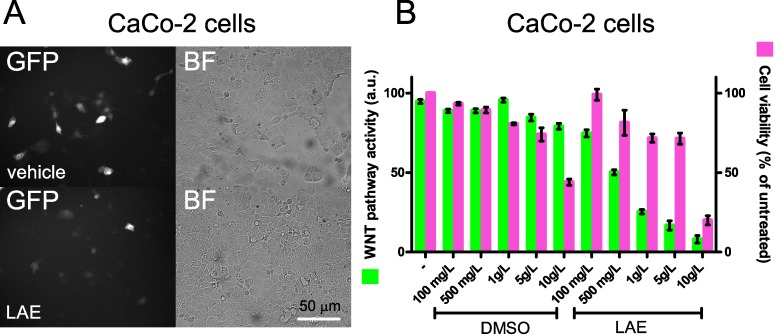
AAE and LAE act as WNT inhibitors in CaCo-2 cells. (**A**) Activity of the WNT reporter construct TCF-wt GFP in CaCo-2 cells cultivated for 48 h in the presence or in the absence of LAE (400 mg/L) (representative of five experiments) (BF = Bright Field; scale bar is shown); (**B**) WNT pathway activity (green bars) and cell viability (magenta bars) of CaCo-2 cells transfected with TCF-wt GFP and cultivated in the absence (−) or in the presence of the indicated concentration of LAE (or of the corresponding dilution of DMSO). Values on the left axes indicate changes in GFP expression (a.u.). Values on the right axes indicate changes in cell viability expressed as percentage of untreated cells (−).

The suitability of LAE and AAE for APC treatment was further proved in vitro, by testing the WNT inhibitory activity of AAE and LAE on CaCo-2 cells. This colon cancer cell line presents a mutation in the APC gene and is commonly used as in vitro cell culture prototype for FAP cells [36,37]. Despite presenting a mutation in APC, CaCo-2 cells are heterogeneous in regard to the WNT pathway activity. This is due to the strong adhesion established among neighbor cells. Cadherins sequester β-catenin at the plasma membrane of the cells, avoiding its translocation to the nucleus (Figure S3). CaCo-2 cells were transiently transfected with TCF-wt GFP (Figure 4A). Thanks to the GFP reporter construct, the small percentage of WNT active cells can be easily followed (Figure 4A). Upon treatment with AAE or LAE (400 mg/L, 48 h), GFP expression decreased in CaCo-2 cells (Figure 4A,B), indicating that the extracts efficiently inhibited the WNT pathway in these cells. Similar to what we measured in HEK293 cells, the extract affected the viability of the cells at dilutions higher than 5.0 g/L, (Figure 4B).

### 3.5. WNT Inhibitory Activity of LAE and AAE on Human Biopsies

**Figure 5:**
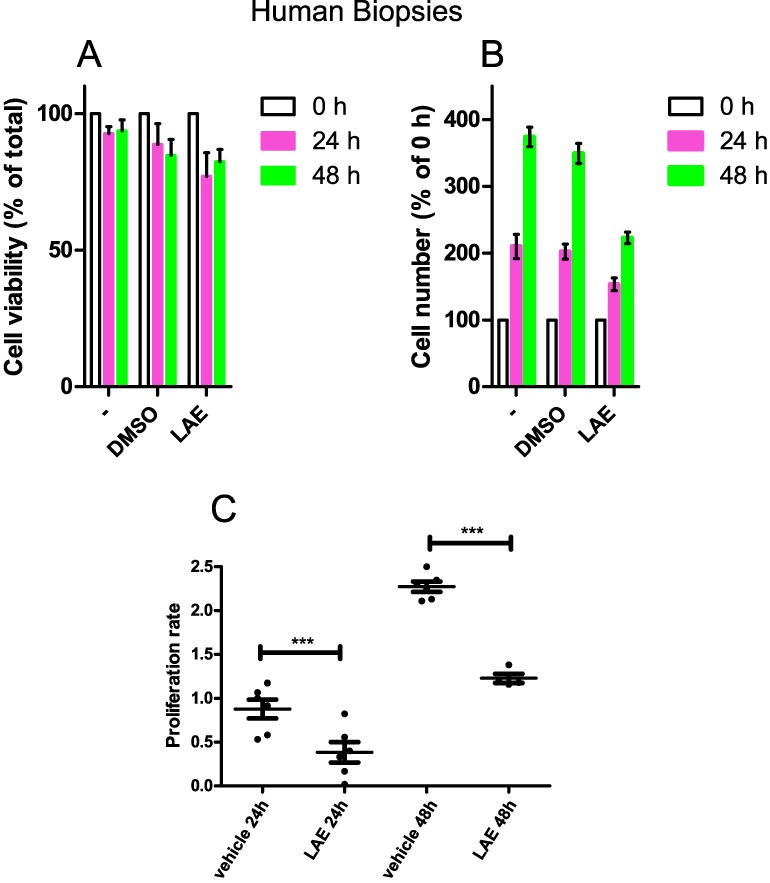
AAE and LAE affect ex vivo cultures of cells carrying FAP mutations. Cell viability, cell number and proliferation rate of human colonic biopsies cultured for 24 h and 48 h in a culture medium supplemented with LAE (400 mg/L) or with vehicle (DMSO). Values are expressed as mean ± SEM (*n* = 9). *** *p* < 0.05.

Finally, AAE and LAE were tested in an ex vivo system of FAP cells. Human colon biopsies were cultured in vitro soon after their resection from FAP patients (Figure 5). In unsupplemented media, these ex vivo samples survived and duplicated for up to two days (Figure 5A), and then underwent growth arrest. Treatment with AAE or LAE resulted in a decreased proliferation rate and survival of the ex vivo cultures (Figure 5A–C). The extracts arrested proliferation and induced cell death at dilutions of 400 mg/L and 5 g/L, respectively.

### 3.6. WNT Inhibitory Activity of Food Grade LAE

**Figure 6:**
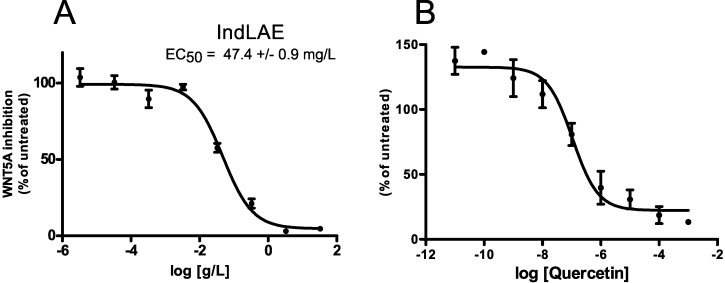
WNT inhibitory activity of IndLAE and quercetin. (**A**) The dose-response curve represents Ind(LAE) modulation of WNT/β-catenin pathway. Values are reported as mean ± SD (*n* = 3). The EC_50_ value for Ind(LAE) is shown on the graph and is reported as mean ± SEM (*n* = 5); (**B**) The dose-response curve represents quercetin modulation of the WNT pathway in HEK293 cells. Values are reported as mean ± SD (*n* = 3). EC_50_ value for quercetin is reported in the text.

Food-grade manufacturing of fruit extracts can often affect patterns and structures of polyphenols. This is because extraction procedures for alimentary purposes only allow water and ethanol as solvents. Thus, the industry makes use of high temperatures and harsher conditions to increase the yield of extraction. We here tested the WNT inhibitory activity of apple extracts obtained from industrial-scale food-grade preparation of LAE (hereinafter referred as IndLAE). IndLAE presents a WNT inhibitory activity increase compared to LAE (EC_50_ of WNT pathway inhibition were of 47.4 ± 0.9 mg/L) (Figure 6A). A HPLC-DAD analysis of the flavonoid content of the IndLAE did not reveal major changes in the number of polyphenols compared to AAE and LAE (Table 3). However, we realized the presence of a discrete amount of quercetin, a molecule that was absent in the apple extracts, which probably arose from the conversion of quercetin glycosides into their aglycone form. Tested in our WNT inhibitory assay and as already demonstrated [38,39], quercetin had a strong inhibitory activity on the WNT pathway (EC_50_ at 110 ± 5 nM, Figure 6B) compared to the other polyphenols. This suggests that the molecule may contribute to the increased WNT inhibitory activity of the industrial extract.

### 3.7. WNT Inhibitory Activity of LAE after In Vitro Simulated GI-Digestion

**Figure 7:**
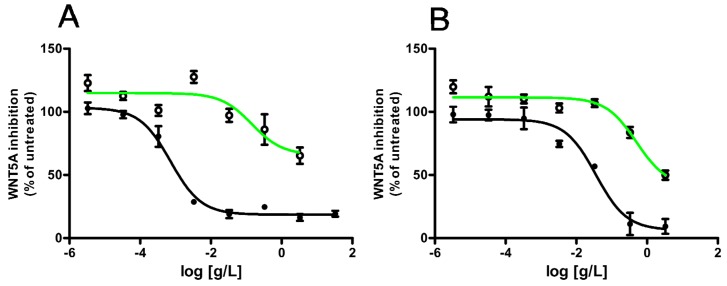
In vitro simulated GI digestion reduces WNT inhibitory activity of AAE and LAE. Dose-response curves for AAE (**A**) and LAE. (**B**) Modulation of the WNT/β-catenin pathway before (black lines) or after in vitro simulated GI digestion (green lines). Values are reported as mean ± SD (*n* = 3).

To achieve WNT inhibitory activity in the colon, apple polyphenolic must be bio-accessible, i.e., they should be extracted from the food matrix, resist to GI-digestion and reach the colon-rectal section of the intestine. However, GI digestion may affect substantially native apple polyphenolic patterns and concentrations as well as induce drastic structural changes of the food constituents [40]. During GI digestion, polyphenols may be further degraded [41] or hydrolyzed, deglycosylated or cleaved by esterases [42]. To measure the bio-accessibility of the WNT inhibitory pool of apple polyphenol, we subjected apple extracts to in vitro simulated digestion [23]. Upon digestion, AAE and LAE (hereinafter referred to as digAAE and digLAE) drastically lost their WNT inhibitory activity (Figure 7A,B). Results of the HPLC-DAD analysis of the polyphenolic contents of digAAE and digLAE are reported in Table 4. As expected, in vitro digestion decreased the overall polyphenolic content of around 40–50%. For both cultivars, digestion converted procyanidins into monomeric catechins, while total amounts of chlorogenic acid, rutin, epicatechin and isoquercetin were drastically reduced. Interestingly, GI digestion reduced the content of phloridzin in AAE but, not in LAE, where the molecule was almost completely resistant to GI. These results indicate that, most likely, the polyphenols ingested by consumption of fresh fruit or fresh fruit extract, will not reach the colon-rectal segment of the intestinal tract and strongly points towards the encapsulation of the extract in gastro-resistant tablets as an essential requirement to preserve apple extract WNT inhibitory activity.

## 4. Discussion

Polyphenol consumption has been related to several health beneficial effects, such as reduced overall mortality [43], incidence of cancer [10] and cardiovascular diseases [12,13,20,44]. The diet is the principal human source of polyphenolic compounds. Polyphenol-rich foods include fruits, vegetables, whole grains, nuts and olive oil.

We here showed that apple extracts from both Annurca (AAE) and Limoncella (LAE) are endowed with inhibitory activity toward WNT/β-catenin signaling, an intracellular pathway involved in many forms of cancer. Moreover, we showed that both the extracts are potentially suitable for the treatment of FAP, a WNT related disease. In this syndrome, a mutation in the protein APC, leads to hyperactivation of the WNT/β-catenin signaling pathway and, in turn, to uncontrolled intestinal cell proliferation and a high risk for the formation of adenocarcinomas. We showed that both AAE and LAE are able to inhibit WNT signaling in cells carrying APC mutations. In both in vitro cultures of cells as well as in ex vivo biopsies of FAP patients, AAE and LAE blocked the proliferation and duplication rates of colon-rectal cancer cells.

AAE and LAE WNT inhibitory activity can be partially ascribed to their polyphenolic content. However, none of the major constituents of their polyphenolic fractions, tested as pure molecules, exerted strong WNT inhibitory activities, suggesting the importance of the whole pool of polyphenols for the activity of the extracts. WNT pathway inhibitory activity has been described for many individual polyphenols. Epigallocatechin gallate (EGCG) [45], isoquercetin [46], anthocyanins [47], theaflavin [48], ellagic acid derivatives [49], caffeoylquinic acids [50] and polymeric polyphenols [51] have all been shown to act as WNT inhibitors in many in vitro cultures of cancer cells. Some of them, like EGCG and ellagic acid, are undetectable in AAE and LAE [24]. We cannot exclude that these could indeed account, alone, for the entire WNT inhibitory activity of the extracts in which they are contained. However, regarding the polyphenols we detected in AAE and LAE, our results are in line with previous reports describing, for these compounds, EC_50_ for WNT pathway inhibition in the micromolar range [35,52,53]. According to this and other studies [54], upon consumption of apples at consumer-relevant concentrations [55,56,57], none of the polyphenols contained in AAE and LAE (even assuming a full bioavailability) would reach active concentrations for WNT pathway inhibition in the colon-rectal segment of the intestine. This is in line with epidemiologic studies that, more and more, show that supplementation with mixtures of bioactive compounds more than with single components, may be effective in exerting biological activity [10,44,58,59,60].

Our preliminary results suggest that the WNT inhibitory activity of AAE and LAE can be ascribed either to the antioxidant activity of the entire polyphenolic mixture or to the effects that the latter may exert on its own components, protecting them from detrimental chemical modifications occurring in physiological environments.

We here showed that food-grade large-scale manufacturing alters the flavonoid composition of LAE, drastically increasing the WNT inhibitory activity of the extract. This could be ascribed to the presence of quercetin, a potent β-catenin inhibitor, absent in the original extract, which probably originated from the conversion of quercetin-glycosides into their aglycone form. This result should encourage researchers to test the activity of the extract after industrial manufacturing. The latter drastically alters the composition of the extract, even if this event, as we have here shown, does not always have to be detrimental to the properties of the extract.

We finally showed that both LAE and AAE lose activity upon in vitro simulated GI digestion as a result of a severe loss of polyphenols. This strongly points towards encapsulation in gastro-resistant tablets (for example enteric coatings) as an essential requirement to preserve apple extract WNT inhibitory activity in the colon-rectal section of the intestinal tract.

## Figures and Tables

**Table 1:** Polyphenolic content of AAE and LAE.

Compound	AAE *	LAE *	PEF (AAE) **	PEF (LAE) **
Chlorogenic Acid	3.9 ± 0.2	4.7 ± 0.1	10.2 ± 0.6	9.7 ± 0.9
(+) Catechin	0.8 ± 0.1	1.3 ± 0.1	3.0 ± 0.2	5.5 ± 0.4
(–) Epicatechin	0.9 ± 0.2	1.3 ± 0.1	2.8 ± 0.1	4.0 ± 0.2
Isoquercetin	1.4 ± 0.1	2.9 ± 0.2	3.4 ± 0.3	2.7 ± 0.3
Rutin	11.2 ± 0.2	0.4 ± 0.3	34.7 ± 0.1	1.7 ± 0.2
Phloridzin	2.1 ± 0.3	1.2 ± 0.5	7.5 ± 0.2	3.0 ± 0.1
Procyanidin B2	1.3 ± 0.1	2.8 ± 0.1	4.1 ± 0.1	10.8 ± 0.1
Phloretin	n.d.	n.d.	n.d.	n.d.
Quercetin	n.d.	n.d.	n.d.	n.d.

Polyphenolic content present in: Annurca Apple Extract (AAE), Limoncella Apple Extract (LAE), a polyphenolic enriched fraction of AAE (PEF(AAE)), a polyphenolic enriched fraction of LAE ((PEF(LAE)). * mg/100 g of Fresh Weight (FW); ** mg/100 mg Dry Weight (DW); n.d., not detected.

**Table 2:** WNT inhibitory activity of pure polyphenols.

Compound	^a^ EC_50_ (M)	^b^ M
Chlorogenic Acid	3.4 ± 0.2 × 10^−6^	≈5.3 × 10^−8^
(+) Catechin	2.5 ± 0.3 × 10^−4^	≈1.8 × 10^−8^
(–) Epicatechin	>>10^−4^	≈2.6 × 10^−8^
Isoquercetin	1.8 ± 0.1 × 10^−6^	≈7.8 × 10^−9^
Rutin	3.1 ± 0.4 × 10^−6^	≈2.6 × 10^−9^
Phloridzin	1.2 ± 0.2 × 10^−5^	≈1.1 × 10^−8^
Procyanidin B2	1.4 ± 0.3 × 10^−6^	≈1.9 × 10^−8^

^a^ EC_50_ of WNT pathway inhibition of the indicated compounds. Values are reported as mean ± SEM (*n* = 3); ^b^ Molarity of the indicated compounds in a solution of LAE, diluted at its EC_50_ of WNT pathway inhibition.

**Table 3:** Polyphenolic content of industrial-scale food-grade LAE.

Compound	Ind(LAE) *
Chlorogenic Acid	8.6 ± 0.1
(+) Catechin	1.4 ± 0.2
(–) Epicatechin	0.7 ± 0.1
Isoquercetin	0.5 ± 0.1
Rutin	2.6 ± 0.3
Phloridzin	7.0 ± 0.4
Procyanidin B2	2.5 ± 0.1
Quercetin	1.2 ± 0.1
Phloretin	n.d.

Polyphenolic content of Industrial Limoncella Extract (IndLAE). * mg/100 g DW; Values are reported as mean ± SD.

**Table 4:** Polyphenolic content of in vitro digested AAE and LAE.

Compound	digAAE *	digLAE *
Chlorogenic Acid	0.9 ± 0.1	0.7 ± 0.1
(+) Catechin	2.5 ± 0.1	2.8 ± 0.1
(–) Epicatechin	0.1 ± 0.1	0.3 ± 0.1
Isoquercetin	0.1 ± 0.1	0.1 ± 0.1
Rutin	0.3 ± 0.2	n.d.
Phloridzin	n.d.	0.3 ± 0.1
Procyanidin B2	n.d.	0.2 ± 0.1
Phloretin	n.d.	n.d.
Quercetin	n.d.	n.d.

Polyphenolic content of: in vitro GI-digested Annurca Apple Extract (digAAE), in vitro GI-digested Limoncella Apple Extract (digLAE); * mg/100 g of DW. Values are reported as mean ± SD.

## Supplementary Materials

The following are available online at http://www.mdpi.com/2072-6643/9/11/1262/s1. Figure S1: Description of the biological platform used to measure the activity of canonical WNT/β-catenin signaling in HEK293 cells, Figure S2: AAE and LAE inhibit WNT/β-catenin signaling in HEK293 cells, Figure S3: WNT/β-catenin pathway activity in CaCo-2 cells.

## References

[1] Familial Adenomatous Polyposis. http://www.orpha.net/consor/cgi-bin/OC_Exp.php?Lng=GB&Expert=733.

[2] Galiatsatos P., Foulkes W.D. (2006). Familial Adenomatous Polyposis.

[3] Stamos J.L., Weis W.I. (2013). The β-catenin destruction complex. Cold Spring Harb. Perspect. Biol..

[4] Kimelman D., Xu W. (2006). β-Catenin destruction complex: Insights and questions from a structural perspective. Oncogene.

[5] Clevers H., Nusse R. (2012). Wnt/β-catenin signaling and disease. Cell.

[6] Krausova M., Korinek V. (2014). Wnt signaling in adult intestinal stem cells and cancer. Cell Signal..

[7] Burgess A.W., Faux M.C., Layton M.J., Ramsay R.G. (2011). Wnt signaling and colon tumorigenesis—A view from the periphery. Exp. Cell Res..

[8] Schepers A., Clevers H. (2012). Wnt signaling, stem cells, and cancer of the gastrointestinal tract. Cold Spring Harb. Perspect. Biol..

[9] He X., Sun L.-M. (2016). Dietary intake of flavonoid subclasses and risk of colorectal cancer: Evidence from population studies. Oncotarget.

[10] Grosso G., Godos J., Lamuela-Raventos R., Ray S., Micek A., Pajak A., Sciacca S., D’Orazio N., Del Rio D., Galvano F. (2017). A comprehensive meta-analysis on dietary flavonoid and lignan intake and cancer risk: Level of evidence and limitations. Mol. Nutr. Food Res..

[11] Ishibashi M. (2015). Natural compounds with Wnt signal modulating activity. Nat. Prod. Rep..

[12] Fazio C., Ricciardiello L. (2014). Components of the Mediterranean Diet with chemopreventive activity toward colorectal cancer. Phytochem. Rev..

[13] Tsao R. (2010). Chemistry and Biochemistry of Dietary Polyphenols. Nutrients.

[14] Vauzour D., Rodriguez-Mateos A., Corona G., Oruna-Concha M.J., Spencer J.P.E. (2010). Polyphenols and Human Health: Prevention of Disease and Mechanisms of Action. Nutrients.

[15] Kern M., Pahlke G., Ngiewih Y., Marko D. (2006). Modulation of key elements of the Wnt pathway by apple polyphenols. J. Agric. Food Chem..

[16] Amado N.G., Fonseca B.F., Cerqueira D.M., Neto V.M., Abreu J.G. (2011). Flavonoids : Potential Wnt/beta-catenin signaling modulators in cancer. Life Sci..

[17] Amado N.G., Predes D., Moreno M.M., Carvalho I.O., Mendes F.A. (2014). Flavonoids and Wnt/β-Catenin Signaling : Potential Role in Colorectal Cancer Therapies. Int. J. Mol. Sci..

[18] Tarapore R.S., Siddiqui I.A. (2012). Modulation of Wnt/β -catenin signaling pathway by bioactive food components. Carcinogenesis.

[19] Fini L., Piazzi G., Daoud Y., Selgrad M., Maegawa S., Garcia M., Fogliano V., Romano M., Graziani G., Vitaglione P. (2011). Chemoprevention of Intestinal Polyps in ApcMin/+ Mice Fed with Western or Balanced Diets by Drinking Annurca Apple Polyphenol Extract. Cancer Prev. Res..

[20] Niedzwiecki A., Roomi M.W., Kalinovsky T., Rath M. (2016). Anticancer efficacy of polyphenols and their combinations. Nutrients.

[21] Scafuri B., Marabotti A., Carbone V., Minasi P., Dotolo S., Facchiano A. (2016). A theoretical study on predicted protein targets of apple polyphenols and possible mechanisms of chemoprevention in colorectal cancer. Sci. Rep..

[22] Tenore G.C., Calabrese G., Stiuso P., Ritieni A., Giannetti D., Novellino E. (2014). Effects of Annurca apple polyphenols on lipid metabolism in HepG2 cell lines: A source of nutraceuticals potentially indicated for the metabolic syndrome. Food Res. Int..

[23] Tenore G.C., Campiglia P., Ritieni A., Novellino E. (2013). In vitro bioaccessibility, bioavailability and plasma protein interaction of polyphenols from Annurca apple (*M. pumila* Miller cv Annurca). Food Chem..

[24] Sommella E., Pepe G., Pagano F., Ostacolo C., Tenore G.C., Russo M.T., Novellino E., Manfra M., Campiglia P. (2015). Detailed polyphenolic profiling of Annurca apple (*M. pumila* Miller cv Annurca) by a combination of RP-UHPLC and HILIC, both hyphenated to IT-TOF mass spectrometry. Food Res. Int..

[25] Tenore G.C., Caruso D., Buonomo G., D’Urso E., D’Avino M., Campiglia P., Marinelli L., Novellino E. (2017). Annurca (*Malus pumila* Miller cv. Annurca) apple as a functional food for the contribution to a healthy balance of plasma cholesterol levels: Results of a randomized clinical trial. J. Sci. Food Agric..

[26] D’Abrosca B., Pacifico S., Cefarelli G., Mastellone C., Fiorentino A. (2007). “Limoncella” apple, an Italian apple cultivar: Phenolic and flavonoid contents and antioxidant activity. Food Chem..

[27] Panzella L., Petriccione M., Rega P., Scortichini M., Napolitano A. (2013). A reappraisal of traditional apple cultivars from Southern Italy as a rich source of phenols with superior antioxidant activity. Food Chem..

[28] Raiola A., Meca G., Mañes J., Ritieni A. (2012). Bioaccessibility of Deoxynivalenol and its natural co-occurrence with Ochratoxin A and Aflatoxin B1 in Italian commercial pasta. Food Chem. Toxicol..

[29] Mari A., Tedesco I., Nappo A., Russo G.L., Malorni A., Carbone V. (2010). Phenolic compound characterisation and antiproliferative activity of “Annurca” apple, a southern Italian cultivar. Food Chem..

[30] Kamino M., Kishida M., Kibe T., Ikoma K., Iijima M., Hirano H., Tokudome M., Chen L., Koriyama C., Yamada K. (2011). Wnt-5a signaling is correlated with infiltrative activity in human glioma by inducing cellular migration and MMP-2. Cancer Sci..

[31] Jin X., Jeon H.Y., Joo K.M., Kim J.K., Jin J., Kim S.H., Kang B.G., Beck S., Lee S.J., Kim J.K. (2011). Frizzled 4 regulates stemness and invasiveness of migrating glioma cells established by serial intracranial transplantation. Cancer Res..

[32] Stornaiuolo M., La Regina G., Passacantilli S., Grassia G., Coluccia A., La Pietra V., Giustiniano M., Cassese H., Di Maro S., Brancaccio D. (2015). Structure-based lead optimization and biological evaluation of BAX direct activators as novel potential anticancer agents. J. Med. Chem..

[33] Generoso S.F., Giustiniano M., La Regina G., Bottone S., Passacantilli S., Di Maro S., Cassese H., Bruno A., Mallardo M., Dentice M. (2015). Pharmacological folding chaperones act as allosteric ligands of Frizzled4. Nat. Chem. Biol..

[34] Kim S.E., Lee W.J., Choi K.Y. (2007). The PI3 kinase-Akt pathway mediates Wnt3a-induced proliferation. Cell Signal..

[35] Kern M., Tjaden Z., Ngiewih Y., Puppel N., Will F., Dietrich H., Pahlke G., Marko D. (2005). Inhibitors of the epidermal growth factor receptor in apple juice extract. Mol. Nutr. Food Res..

[36] Morin P.J., Sparks B., Korinek V., Barker N., Clevers H., Vogelstein B., Kinzler K.W. (1997). Activation of beta-catenin-Tcf signaling in colon cancer by mutations in beta-catenin or APC. Science.

[37] Sansom O.J., Reed K.R., Hayes A.J., Ireland H., Brinkmann H., Newton I.P., Batlle E., Simon-Assmann P., Clevers H., Nathke I.S. (2004). Loss of Apc in vivo immediately perturbs Wnt signaling, differentiation, and migration. Genes Dev..

[38] Park C.H., Chang J.Y., Hahm E.R., Park S., Kim H.-K., Yang C.H. (2005). Quercetin, a potent inhibitor against beta-catenin/Tcf signaling in SW480 colon cancer cells. Biochem. Biophys. Res. Commun..

[39] Pahlke G., Ngiewih Y., Kern M., Jakobs S., Marko D., Eisenbrand G. (2006). Impact of quercetin and EGCG on key elements of the Wnt pathway in human colon carcinoma cells. J. Agric. Food Chem..

[40] Bouayed J., Hoffmann L., Bohn T. (2011). Total phenolics, flavonoids, anthocyanins and antioxidant activity following simulated gastro-intestinal digestion and dialysis of apple varieties: Bioaccessibility and potential uptake. Food Chem..

[41] Liang L., Wu X., Zhao T., Zhao J., Li F., Zou Y., Mao G., Yang L. (2012). In vitro bioaccessibility and antioxidant activity of anthocyanins from mulberry (*Morus atropurpurea* Roxb.) following simulated gastro-intestinal digestion. Food Res. Int..

[42] Bouayed J., Deußer H., Hoffmann L., Bohn T. (2012). Bioaccessible and dialysable polyphenols in selected apple varieties following in vitro digestion vs. their native patterns. Food Chem..

[43] Grosso G., Micek A., Godos J., Pajak A., Sciacca S., Galvano F., Giovannucci E.L. (2017). Dietary Flavonoid and Lignan Intake and Mortality in Prospective Cohort Studies: Systematic Review and Dose-Response Meta-Analysis. Am. J. Epidemiol..

[44] Wang X., Ouyang Y.Y., Liu J., Zhao G. (2014). Flavonoid intake and risk of CVD: A systematic review and meta-analysis of prospective cohort studies. Br. J. Nutr..

[45] Oh S., Gwak J., Park S., Yang C.S. (2014). Green tea polyphenol EGCG suppresses Wnt/β-catenin signaling by promoting GSK-3β- and PP2A-independent β-catenin phosphorylation/degradation. BioFactors.

[46] di Camillo Orfali G., Duarte A.C., Bonadio V., Martinez N.P., de Araújo M.E.M.B., Priviero F.B.M., Carvalho P.O., Priolli D.G. (2016). Review of anticancer mechanisms of isoquercitin. World J. Clin. Oncol..

[47] de Sousa Moraes L.F., Sun X., Peluzio M.D.C.G., Zhu M.-J. (2017). Anthocyanins/anthocyanidins and colorectal cancer: What is behind the scenes?. Crit. Rev. Food Sci. Nutr..

[48] Sur S., Pal D., Mandal S., Roy A., Panda C.K. (2016). Tea polyphenols epigallocatechin gallete and theaflavin restrict mouse liver carcinogenesis through modulation of self-renewal Wnt and hedgehog pathways. J. Nutr. Biochem..

[49] Ramírez de Molina A., Vargas T., Molina S., Sánchez J., Martínez-Romero J., González-Vallinas M., Martín-Hernández R., Sánchez-Martínez R., Gómez de Cedrón M., Dávalos A. (2015). The ellagic acid derivative 4,4′-di-O-methylellagic acid efficiently inhibits colon cancer cell growth through a mechanism involving WNT16. J. Pharmacol. Exp. Ther..

[50] Taira J., Uehara M., Tsuchida E., Ohmine W. (2014). Inhibition of the β-catenin/Tcf signaling by caffeoylquinic acids in sweet potato leaf through down regulation of the Tcf-4 transcription. J. Agric. Food Chem..

[51] Patel R., Ingle A., Maru G.B. (2008). Polymeric black tea polyphenols inhibit 1,2-dimethylhydrazine induced colorectal carcinogenesis by inhibiting cell proliferation via Wnt/beta-catenin pathway. Toxicol. Appl. Pharmacol..

[52] Amado N.G., Predes D., Fonseca B.F., Cerqueira D.M., Reis A.H., Dudenhoeffer A.C., Borges H.L., Mendes F.A., Abreu J.G. (2014). Isoquercitrin suppresses colon cancer cell growth in vitro by targeting the Wnt/β-catenin signaling pathway. J. Biol. Chem..

[53] Kim H., Seo E.-M., Sharma A.R., Ganbold B., Park J., Sharma G., Kang Y.-H., Song D.-K., Lee S.-S., Nam J.-S. (2013). Regulation of Wnt signaling activity for growth suppression induced by quercetin in 4T1 murine mammary cancer cells. Int. J. Oncol..

[54] Fantini M., Benvenuto M., Masuelli L., Frajese G.V., Tresoldi I., Modesti A., Bei R. (2015). In vitro and in vivo antitumoral effects of combinations of polyphenols, or polyphenols and anticancer drugs: Perspectives on cancer treatment. Int. J. Mol. Sci..

[55] Crozier A., Del Rio D., Clifford M.N. (2010). Bioavailability of dietary flavonoids and phenolic compounds. Mol. Aspects Med..

[56] Grosso G., Stepaniak U., Topor-Madry R., Szafraniec K., Pajak A. (2014). Estimated dietary intake and major food sources of polyphenols in the Polish arm of the HAPIEE study. Nutrition.

[57] Zamora-Ros R., Knaze V., Rothwell J.A., Hémon B., Moskal A., Overvad K., Tjønneland A., Kyrø C., Fagherazzi G., Boutron-Ruault M.C. (2016). Dietary polyphenol intake in europe: The european prospective investigation into cancer and nutrition (EPIC) study. Eur. J. Nutr..

[58] Tulp M., Bruhn J.G., Bohlin L. (2006). Food for thought. Drug Discov. Today.

[59] Visioli F., Bogani P., Grande S., Galli C. (2005). Mediterranean food and health: Building human evidence. J. Physiol. Pharmacol..

[60] La Vecchia C. (2009). Association between Mediterranean dietary patterns and cancer risk. Nutr. Rev..

