# Supplementary material for: WNT Inhibitory Activity of Malus Pumila miller cv Annurca and Malus domestica cv Limoncella Apple Extracts on Human Colon-Rectal Cells Carrying Familial Adenomatous Polyposis Mutations
Source: Nutrients. 2017 Nov 18;9(11):1262. doi: 10.3390/nu9111262 (PMC5707734; doi:10.3390/nu9111262)
Supplement: Supplementary file 1 [file nutrients-09-01262-s001.pdf]

SUPPLEMENTARY DATA

WNT inhibitory activity of *Malus Pumila Miller* cv Annurca and *Malus Domestica* cv Limoncella apple extracts on human colon-rectal cells carrying Familial Adenomatous Polyposis mutations.

Gennaro Riccio [1], Maria Maisto [1], Sara Bottone [1], Nadia Badolati [1], Giovanni Battista Rossi[2], Gian Carlo Tenore [1], Mariano Stornaiuolo[1,*] and Ettore Novellino [1,*]

[1] Department of Pharmacy, University of Naples Federico II, 80131 Naples, Italy;(G.R.: genriccio@gmail.com ; M.M.: maria.maisto@unina.it; S.B.: sara.bottone@unina.it; N.B. : badolatin@gmail.com; G.C.T.: giancarlo.tenore@unina.it)
[2] Gastroenterology and Gastrointestinal Endoscopy Unit, Istituto Nazionale Tumori -IRCCS- Fondazione G. Pascale, 80131 Naples, Italy; g.rossi@istitutotumori.na.it

* Correspondence: mariano.stornaiuolo@ unina.it; Tel.: +39-081-670117
ettore.novellino@unina.it; Tel.: +39-081-678643

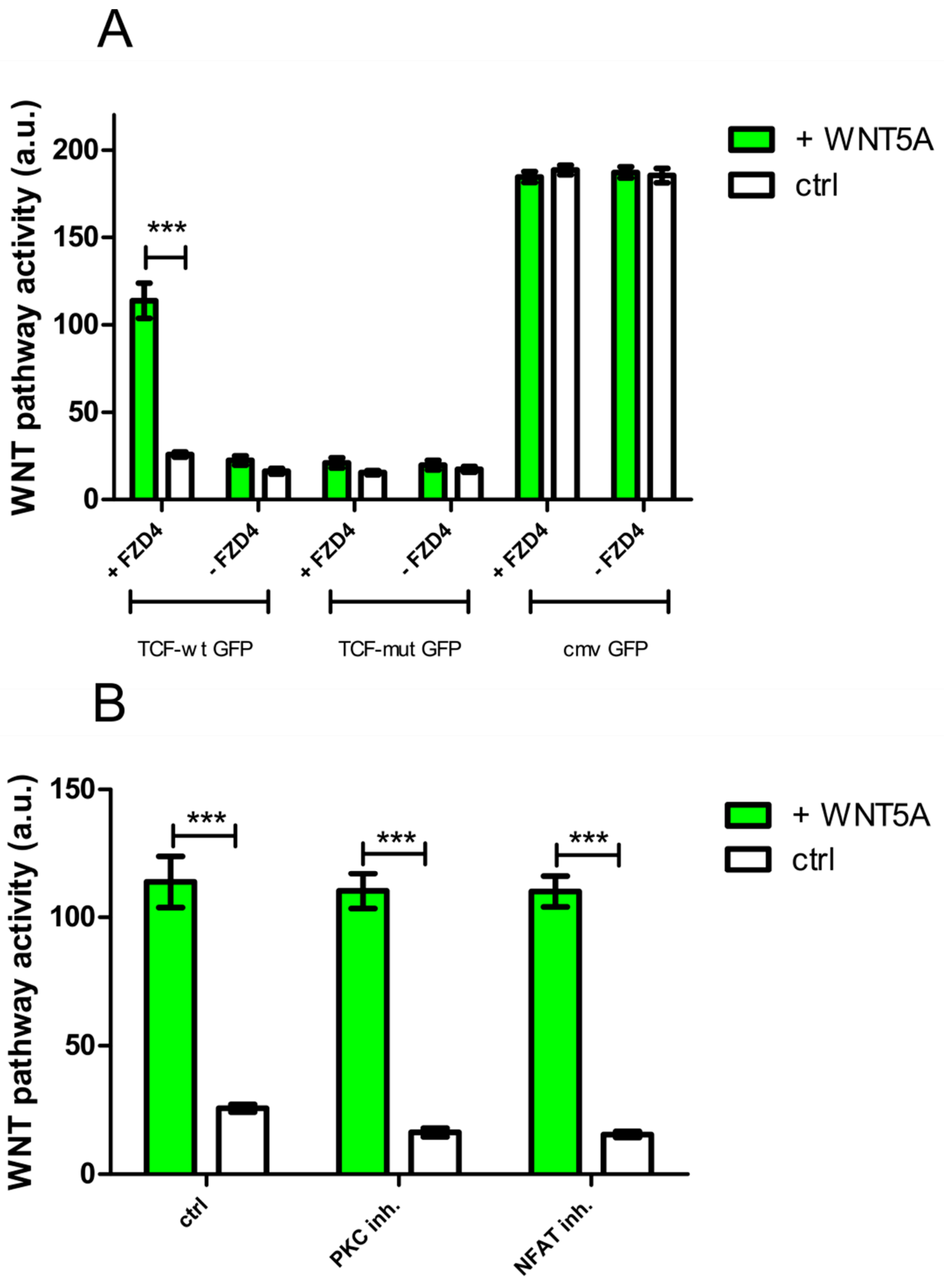

Figure S1. Setting up of the biological platform used to measure canonical WNT/β-catenin pathway activity in HEK293 cells. **(A)** HEK293 cells were transfected with cDNAs coding for the WNT reporter construct TCF-wt GFP and the WNT receptor FZD4. Activation of the pathway by WNT5A (a FZD4 agonist) induces expression of GFP. The histogram shows the increase in WNT pathway activity upon treatment with WNT5A, in the presence or in the absence of the WNT receptor FZD4. The measured increase in GFP expression upon treatment with WNT5A relies on the presence of a functional TCF/LEF sequence. Indeed, WNT5A does not increase GFP expression in cells transfected with a mutant WNT reporter GFP construct (TCF-mut GFP) or in cells transfected with a constitutively expressed GFP (cmv GFP). **(B)** Effect of NFAT and PKC inhibitors on the WNT pathway activity elicited by WNT5A. Values are reported as mean +/- SEM (n=5). *** $P<0.05$.

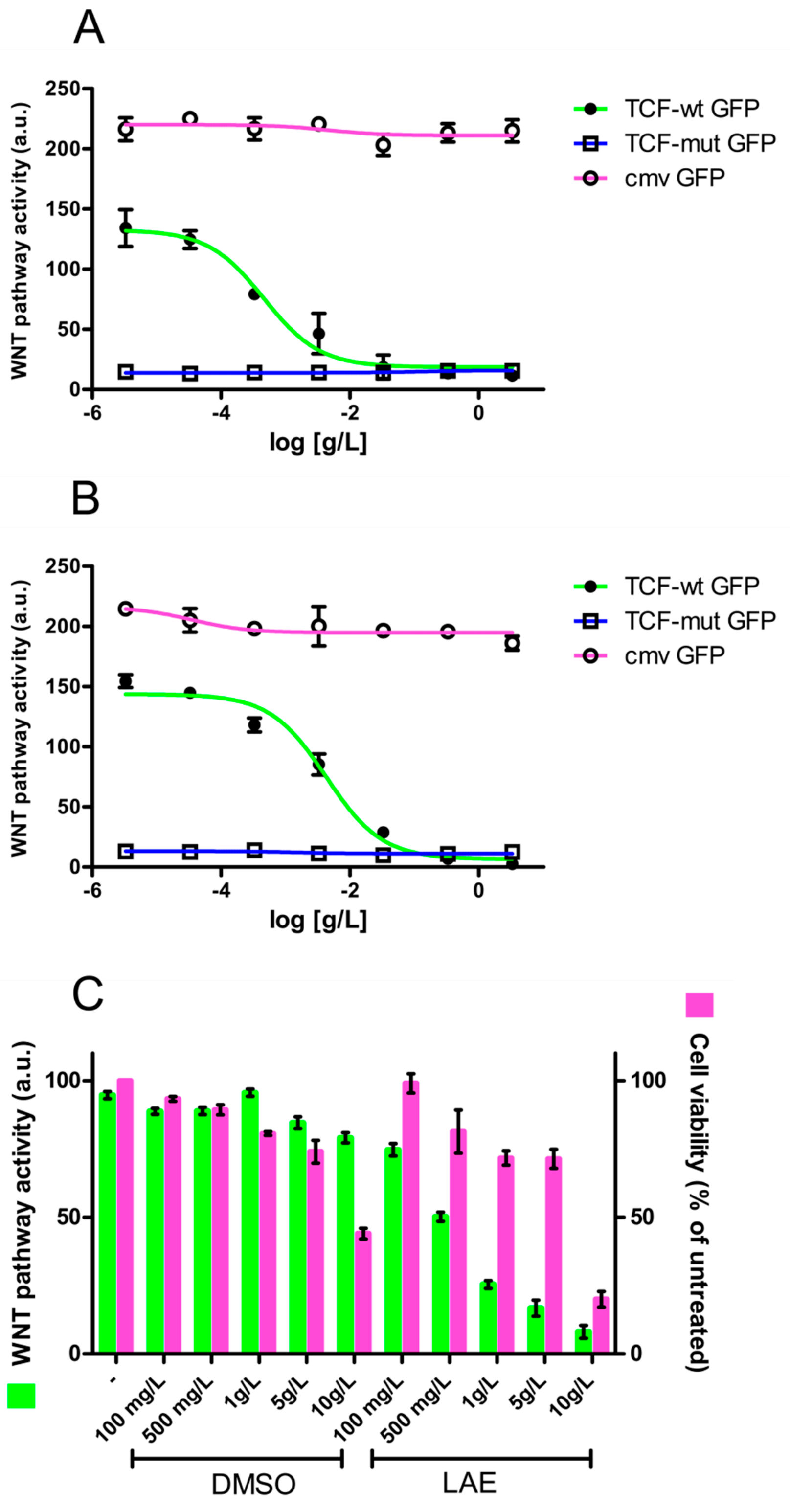

Figure S2. AAE and LAE inhibit WNT/β-catenin in HEK293 cells. (A-B) Dose-response curves represent AAE **(A)** and LAE **(B)** modulation of WNT/β-catenin pathway in HEK293 cells co-expressing FZD4 and either TCF-wt GFP (green curve), TCF-mut GFP (blue curve) or cmv GFP (magenta curve). Values indicate changes in GFP expression. **(C)** WNT pathway activity (green bars) and cell viability (magenta bars) of HEK293 cells coexpressing FZD4 and TCF-wt GFP and cultivated in the absence (-) or in the presence of the indicated concentration of LAE (or of the corresponding dilution of DMSO). Values on the left axes indicate changes in GFP expression (a.u.). Values on the right axes indicate changes in cell viability expressed as percentage of untreated cells (-).Values are reported as mean ± SD (*n* = 3 replicates).

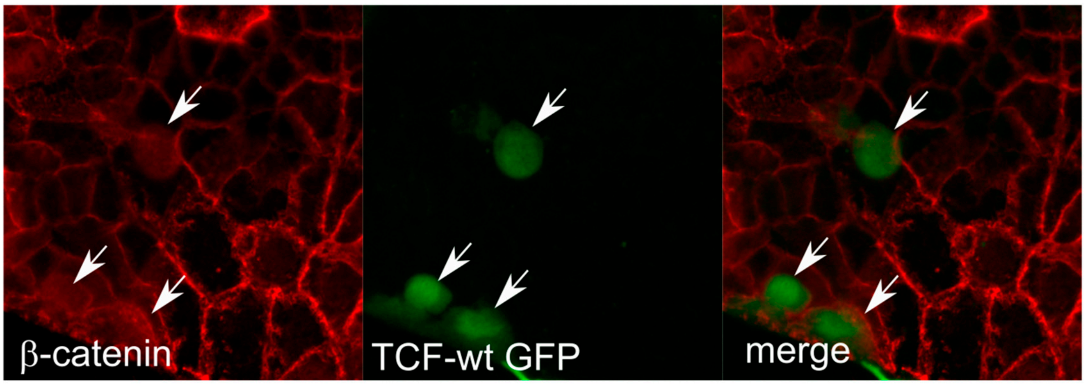

Figure S3. WNT/β-catenin pathway activity in CaCo-2 cells. Non-confocal immunofluorescence showing the intracellular localization of β-catenin in CaCo-2 cells. β-catenin (red channel) is localized on the Plasma Membrane in almost all the cell population. Only a small percentage of cells (here indicated by the arrows) present β-catenin localized in the cytoplasm and in the nucleus. As shown by the activity of the construct TCF-wt GFP (green channel), these cells present an active WNT pathway.
